# The Relationship of Diet Quality with Proportion of Daily Energy Contributed by Sandwiches Varies by Age over Adulthood in Racially and Socioeconomically Diverse Adults

**DOI:** 10.3390/nu12092807

**Published:** 2020-09-13

**Authors:** Marie Fanelli Kuczmarski, May A. Beydoun, Nancy Cotugna, Elizabeth Schwenk, Michele K. Evans, Alan B. Zonderman

**Affiliations:** 1 Department of Behavioral Health and Nutrition, University of Delaware, 021 CSB, 26N College Ave., Newark, DE 19716, USA; ncotugna@udel.edu (N.C.); bschwenk@udel.edu (E.S.); 2 Laboratory of Epidemiology and Population Sciences, National Institute on Aging, NIH, 251 Bayview Blvd. Suite 100, Baltimore, MD 21224-6825, USA; baydounm@mail.nih.gov (M.A.B.); evansm@grc.nia.nih.gov (M.K.E.); zondermana@gmail.com (A.B.Z.)

**Keywords:** diet quality, diet, healthy eating index, African American, urban adults, older adults, adults

## Abstract

Sandwiches are considered a staple in diets of United States adults. Previous research with Healthy Aging in Neighborhoods of Diversity across the Life Span study participants revealed that 16% consume a sandwich dietary pattern providing with 44% of their daily energy. Yet, little is known about the effect of sandwiches on diet quality over time. The study objectives were to determine the relationship of energy contributed by sandwiches to diet quality in this socioeconomically and racially diverse sample categorized by age (<50 years and ≥50 years at baseline) and to describe patterns of sandwich consumption over ~12 years. The analyses included a series of linear mixed-effects regression models, with age as the time variable centered at 50 years. In each model, the main outcome was Healthy Eating Index-2010 score with up to three scores, while the main predictor was % total energy from sandwiches (0, >0–20%, >20%) measured concurrently at each visit. Diet quality of older men with income <125% poverty improved over time for those consuming >0–20% and >20% energy from sandwiches compared to young women with incomes >125% poverty who were non-reporters of sandwiches (β ± SE: 10.93 ± 5.27, *p* = 0.01; 13.11 ± 4.96, *p* = 0.01, respectively). The three most common sandwich types reported, in descending order, were cold cuts, beef, and poultry.

## 1. Introduction

Most food historians attribute the invention of the sandwich, a bread-enclosed convenience food, to John Montagu, fourth Earl of Sandwich (1718–1792) [1]. In the United States (US), approximately 50% of all adults consume one or more sandwiches on any given day [2]. Scientific publications about patterns of sandwich consumption and diet quality are limited in contrast to the examples of the lay press assessment of sandwich intake. An article in an English newspaper, the Guardian, by Belam noted cheese sandwiches and ham sandwiches were among the favorite British lunchtime sandwiches [3]. Approximately 33% of Britons surveyed by Whole Foods Market ate the same lunch daily and half had been doing the same thing for 6 years. The question arises, does repetitive eating yield healthful habits and high-quality diets? Perhaps so, given the results of a review of observation studies by de Oliveira Otto and colleagues [4] which found greater dietary diversity to be associated with suboptimal eating patterns.

The Dietary Guidelines for Americans, 2015–2020, emphasize adherence to a healthful eating pattern across the life span [5]. Li and colleagues found that a healthful lifestyle, defined by 5 low-risk lifestyle factors, led to longer life expectancy for both sexes [6]. The factors were: high diet quality score [upper 40%], moderate alcohol intake, never smoking, body mass index of 18.5 to 24.9 kg/m^2^, and ≥30 min/day of moderate to vigorous physical activity. The upper 40% of diet quality score would be represented by a score of 60 or higher for the Healthy Eating Index (HEI). The mean HEI-2015 scores for Americans based on the What We Eat in America (WWEIA), National Health and Nutrition Examination Survey (NHANES), 2015–2016 were 58.3 for those 18–64 years of age and 64.0 for those 65+ years [7]. It appears that older US individuals, compared to adults of 18–64 years, have better compliance to dietary recommendations.

Sandwiches are major contributors to sodium in the diets of American adults [8]. They can also contribute energy from saturated fats, which should be limited according to the Dietary Guidelines for Americans [5]. However, depending on the bread and ingredients of the sandwich they can contribute fiber and micronutrients limited in the diet [2,9] Since research regarding the relationship between sandwich consumption and diet quality is limited [10], the primary study objective was to determine the association of the percent of daily energy contributed by sandwiches to diet quality in a socioeconomically and racially diverse sample categorized by age. A second aim was to describe their patterns of sandwich consumption. 

## 2. Methods

### 2.1. Sample

Participants, urban-dwelling African American and White adults, were from three waves of the Healthy Aging in Neighborhoods of Diversity across the Life Span (HANDLS) study (*n* = 3720). Evans and colleagues have written a detailed description of this study [11]. The baseline study, referred to as visit 1 (or v_1_) in this article, was initiated in 2004 and completed in 2009. The first follow-up wave, v_2_, was conducted between 2009 and 2013; and the second follow-up wave, v_3_, was done between 2013 and 2017. At v_1_, participants were asked their self-reported household income for the past 12 months which was dichotomized into <125% and >125% of the 2004 Health and Human Services poverty guidelines [12] and will be referred to as < or >125% poverty status in this article. The 125% dichotomization was set since 125% above the poverty line seemed to capture the minimum reasonable household income based on family size for the cost of living in Baltimore in 2004. Demographic characteristics of the sample by visit are presented in Table 1. All participants provided written informed consent at each wave following their access to a protocol booklet in layman’s terms and a video describing all procedures. They were compensated monetarily. The study protocol was approved by the human Institutional Review Boards at MedStar Health Research Institute, the National Institute of Environmental Health Sciences, National Institutes of Health, and the University of Delaware.

### 2.2. Dietary Collection Method

Dietary intake measures were included in our analyses at v_1_, v_2_, and v_3_. For all visits, food intake was collected over 2 days by trained interviewers using the US Department of Agriculture (USDA) Automated Multiple Pass Method (AMPM) [13,14]. This method, along with visual aids used for portion estimation, are described elsewhere [15]. All interviewers completed a 3-day training and also periodic refresher trainings during the year. Except for v_1_ where both interviews were done in-person, at v_2_ and v_3_, the first recall was done in-person and the second recall was done by telephone. Of the 3720 baseline participants, two 24-h recalls were collected from 2177 adults at v_1_, 2140 at v_2_, and 2066 at v_3_.

### 2.3. Food Coding

For this study, the definition of *sandwich* included not only sandwiches in the dietary data represented by a single food code but also those items represented by two or more food codes that were linked and identified as a sandwich combination. Single food code sandwiches were mostly fast food items. Each sandwich or ingredient that comprised a sandwich was assigned an 8-digit code from the Food and Nutrient Database for Dietary Studies, matching the date of the wave to the most appropriate database [16]. The codes were assigned by both the USDA Survey Net data processing system and trained dietary coders. Food descriptions and quantity data collected for each food in the AMPM along with information such as the time, eating occasion, and combination codes were imported into Survey Net, a computer-assisted food coding system [14]. This information was used to aggregate ingredients of sandwiches. 

For sandwich combination items, the energy and nutrients provided by each ingredient were aggregated by the day, time, and eating occasion for each sandwich within a recall day. The majority of reported sandwiches had 2 slices of bread or the equivalent, such as a roll. However, on occasion persons reported a sandwich with one bread slice, for example, a hot dog wrapped in one slice of bread. All sandwiches were assigned a food group number based on the primary filling. There was a total of 10 groups—peanut butter, egg, cheese, beef, poultry, fish, pork, hot dog, fruit and/or vegetable, and cured and processed meats. The cured and processed meat group included sausage, bacon, and deli/luncheon meats. Egg sandwiches were defined as all sandwiches containing egg, even if they also contained some meat or cheese. Cheese sandwiches were defined as sandwiches with only cheese as the primary filling. They did not contain any meat, poultry, fish, or meat alternatives. 

To calculate the energy contributed by sandwiches to a day’s total, for each recall day, the energy provided by the sandwich was divided by the day total for energy and this ratio was multiplied by 100. 

### 2.4. Eating Occasions

During the dietary recall, participants were asked to identify the eating occasion from the following choices: breakfast, lunch, brunch, dinner, supper, snack, drink, extended consumption. For this study, responses for brunch and lunch, as well as for dinner and supper, were combined. 

### 2.5. Source of Sandwiches/Ingredients

For each food reported, participants were asked “Where did you get this (Name of food) or most ingredients for this (Name of food)”. There was a total of 28 source options. For this study, only responses for 3 sources most commonly reported were used—store, fast food restaurant, and other restaurant. Store was calculated based on responses from 3 categories—grocery/supermarket, store-convenience type, and store-no additional information, while other restaurant was based on restaurant with waiter/waitress, bar/tavern, restaurant-no additional information.

### 2.6. Healthy Eating Index 2010 (HEI-2010)

The HEI-2010 was used to evaluate overall diet quality and evaluates compliance to the *Dietary Guidelines for Americans* [5,17]. The basic steps for calculating the HEI-2010 component and total scores and statistical codes for 24-h dietary recalls were provided on The National Cancer Institute’s Applied Research website [18]. A detailed description of the procedure used for this study is available on the HANDLS website [19]. For each visit, component and total HEI-2010 scores were calculated for each recall day and were averaged to obtain the mean for both days combined.

### 2.7. Statistical Analyses

For this study, participants were categorized into two age groups, “younger” defined as those <50 years at v_1_ and “older” defined as ≥50 years at v_1_. The analyses testing the main hypotheses included a series of linear mixed-effects regression models, with age as the time variable centered at 50 years (*Age_50_*) in decade units [20]. We hypothesized that sandwich consumption would be associated with lower diet quality. In each model, the main outcome was HEI-2010 total score with up to 3 repeats at v_1_, v_2_, and v_3_, while the main predictor was % total energy from sandwiches measured concurrently at each visit. All models incorporated *Age_50_*, and 2-way interaction terms between exposure or covariates with *TIME.* The models adjusted for v_1_ HEI-2010, as well as annualized HEI change with age for potentially confounding covariates. These covariates (listed under the *Covariates* section) included age grouped as (<50 years: “younger” vs. ≥50 years: “older”), sex, race, and poverty status. The two-way interaction terms exposure × *Age_50_* are interpreted as the effects of exposures (net of covariates) on the slope or annual rate of change in HEI-2010. The main effects of exposures, also included in the mixed-effect linear regression models, allowed us to examine the net exposure effect on baseline HEI-2010 total score, i.e., the cross-sectional exposure-outcome association, controlling for v_1_ covariates. Random effects were added to *Age_50_* and the intercept in the model, assuming an unstructured variance-covariance matrix. The mean number of repeats/participants in our present analysis was 2.2. Further addition of a random effect to the time-dependent exposure did not alter the model significantly and thus, this random effect was excluded. In each model, we assumed missingness of outcomes to be at random (Supplementary methods) [21]. In addition to 2-way interaction terms between *Age_50_* and exposures/covariates, other interaction terms were added to examine heterogeneity of the cross-sectional and longitudinal effects of the exposure on the outcome across age group, sex, race, and poverty status. Those were up to 6-way interactions and included 2- to 4-way interactions between exposures and covariates as well as 3- to 6-way interactions between exposures, covariates, and *Age*_50_. 

Given that the main exposure was coded as a 3-level categorical variable, contrasts were made between non-sandwich reporters (coded as “0”) with the two levels of sandwich eaters (1: “>0–20% of total energy”; 2: “>20% of total energy”). The cutoff of 20 represents the median energy contributed from sandwiches for those who reported sandwich consumption. From this complex multiple mixed-effects linear regression model, differences in the fixed effects of exposure on baseline and change in HEI-2010 across groups were of primary interest and were reported at type I error of 0.05 ((3-way (e.g., exposure by sex; exposure by age group etc.) up to 6-way interaction terms (i.e., exposure by *Age_50_* by Age group by sex by race by poverty status)). The predictive margins from these models were then estimated and plotted as lines to examine the effects of each exposure level on the HEI-2010 outcome at age 50 years and change in this outcome for each age group, sex, race, and poverty status category. All categorical variables are coded as 0/1 or are a series of dummy variables with one common referent and age was centered at 50. The prediction of HEI-2010 at age of 50 years within all these categories based on the same mixed-effects linear regression model was also presented using a series of radar plots which allows one to contrast point estimates across those groups in a visually efficient manner. Full model results are in Supplementary Table S1. Analyses were performed with Stata, Release 16 [22].

For descriptive analysis of the types of sandwiches consumed, only participants who completed both recalls at all three visits (*n* = 1213) were used. Logistic regression was used to compare sociodemographic factors of these individuals to HANDLS v_1_ sample of 3720 who did not complete recalls at each visit. More women and individuals with incomes >125% of the poverty threshold completed two recalls at all 3 visits. There were no statistically significant differences for race or age at v_1_. The statistical significance for all analyses was established using *p* < 0.05. 

## 3. Results

### 3.1. Association of Energy Contributed by Sandwiches to Diet Quality

The mean percent energy contributed by sandwiches for the younger age group ranged from approximately 15% to 21%, in comparison to 16% to 25% for the older group (Table 2). For the group categorized as consuming >0% to 20% of energy from sandwiches their mean (±SE) energy from sandwiches was 12.71 ± 0.10%; for the >20% energy group, their mean energy intake from sandwiches was 33.94 ± 0.24%. The mean ± SE for the HEI-2010 score for the entire sample was 45.80 ± 0.20 out of 100. The HEI-2010 scores of sandwich consumers categorized by race, sex, poverty status, and age are shown in Table 2. These mean HEI-2010 scores of sandwich consumers ranged from 40.09 to 50.11 out of 100. The mean highest score was found for AA women who were ≥50 years with income ≥125% poverty while the lowest score was observed for White men who were < 50 years with income <125% poverty (Table 2).

**Figure 1:**
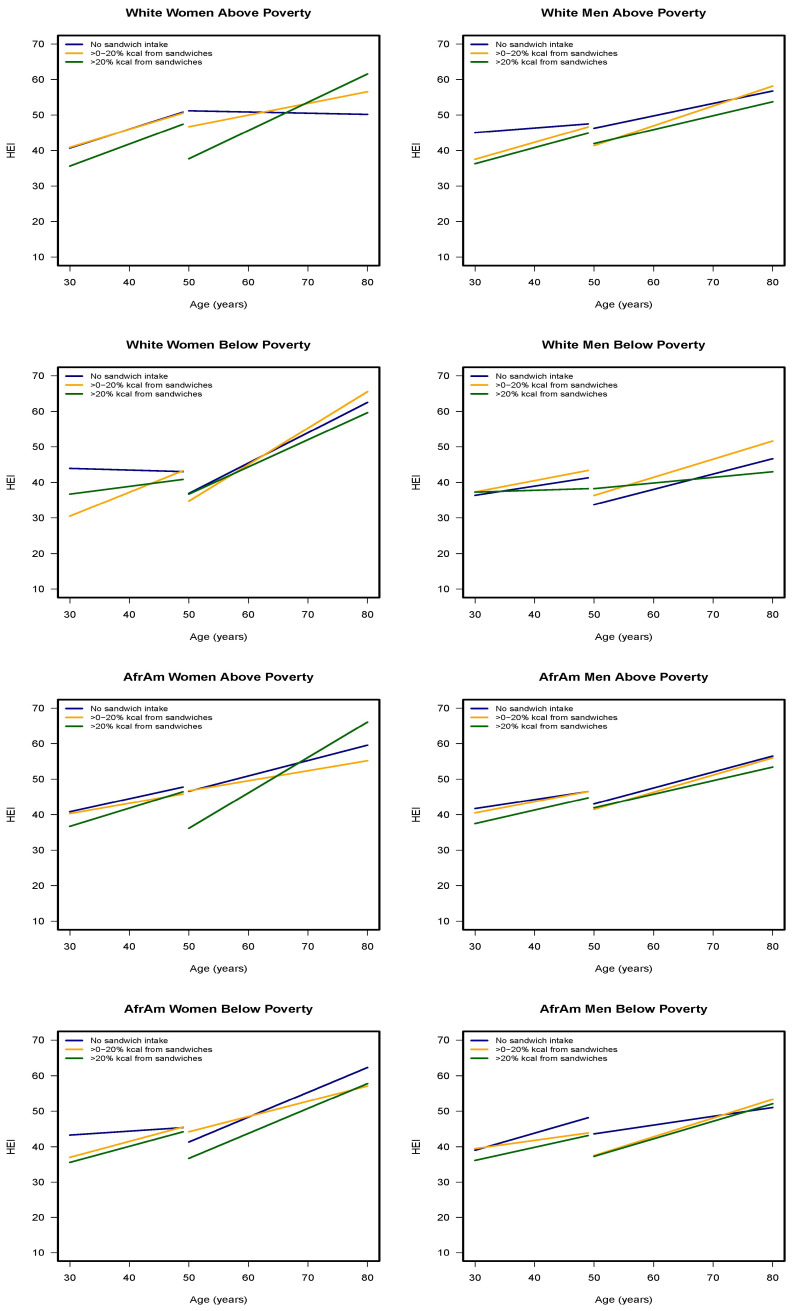
Healthy Eating Index (HEI) scores across three visits of the HANDLS study (2004–2017). For African American and White men and women by initial age cohort (<50 or ≥50 years at visit one) and by poverty (<125% or >125% poverty threshold) for three levels of energy intake (0%, >0–20%, >20% kcal) contributed by sandwiches. HANDLS-Healthy Aging in Neighborhoods of Diversity across the Life Span.

Figure 1 presents the change in HEI-2010 scores over time for sandwich energy group by age, race, sex, and poverty status groups. The mixed-effects regression analyses found two 5-way, four 4-way, three 3-way and one 2-way significant interactions involving energy from sandwiches with HEI-2010 as the outcome (Table S2). The two significant 5-way interactions were age group (older vs. younger) *x* sex (men vs. women) *x* poverty status (<125% vs. >125%) *x* age-centered *x* sandwich energy (<0–20% vs. 0%) (β ± SE: 10.93 ± 5.27, *p* = 0.01) and sandwich energy (>20% vs. 0%) (13.11 ± 4.96, *p* = 0.001). These interactions suggest improvement in diet quality over time for older men with income below poverty who consumed sandwiches compared to younger women with income above poverty who did not report sandwich consumption. Two 3-way interactions, sandwich energy (>0–20% vs. 0) *x* poverty status (<125% vs. >125%) *x* age-centered (7.44 ± 3.10, *p* = 0.01) and sandwich energy (>20% vs. 0) *x* age group (older vs. younger) *x* age-centered (7.40 ± 2.79, *p* = 0.001) also revealed improvement with diet quality over time when compared to non-sandwich reporters. In contrast, a significant 4-way interaction revealed that the diet quality worsened over time for older adults with incomes below poverty who were consuming greater than 20% of their energy intake from sandwiches in comparison to younger adults above poverty to those who did not report sandwich consumption (−10.95 ± 4.33, *p* = 0.01). This association was also found for those who consumed >0–20% of energy from sandwiches (−9.33 ± 4.54, *p* = 0.01). Additionally, over time, diet quality worsened for older men who consumed >20% of energy from sandwiches in comparison to young women who reported no sandwich consumption (−10.22 ± 4.09, *p* = 0.01). The 2-way interaction of sandwich energy (>20% vs. 0) *x* age group (older vs. younger) (−10.21 ± 3.14, *p* = 0.001) indicated their diet quality was less than that of younger adults who did not report sandwiches (Table S1).

The results of the slope analyses revealed there were improvements in diet quality as evidenced by significant and positive change in the slopes of the HEI-2010 scores over time for most groups, regardless of sandwich consumption category. None of the slopes changed significantly for white men who did not report sandwich consumption. The magnitudes in slope change were higher for women than men (Table S2). Older white women with incomes <125% poverty who consumed >0–20% of their daily energy intake from sandwiches and older African American women, with incomes >125% poverty who consumed >20% of their energy from sandwiches experienced the greatest positive change in HEI-2010 scores over time (Slope of 10.08 and 10.03, respectively). Older white women with incomes >125% poverty who consumed >20% of their daily energy intake from sandwiches also had positive steep slope change in HEI-2010. As shown in Figure 1, the diet quality of these groups exceeded that of women who did not report eating sandwiches over time. 

**Figure 2:**
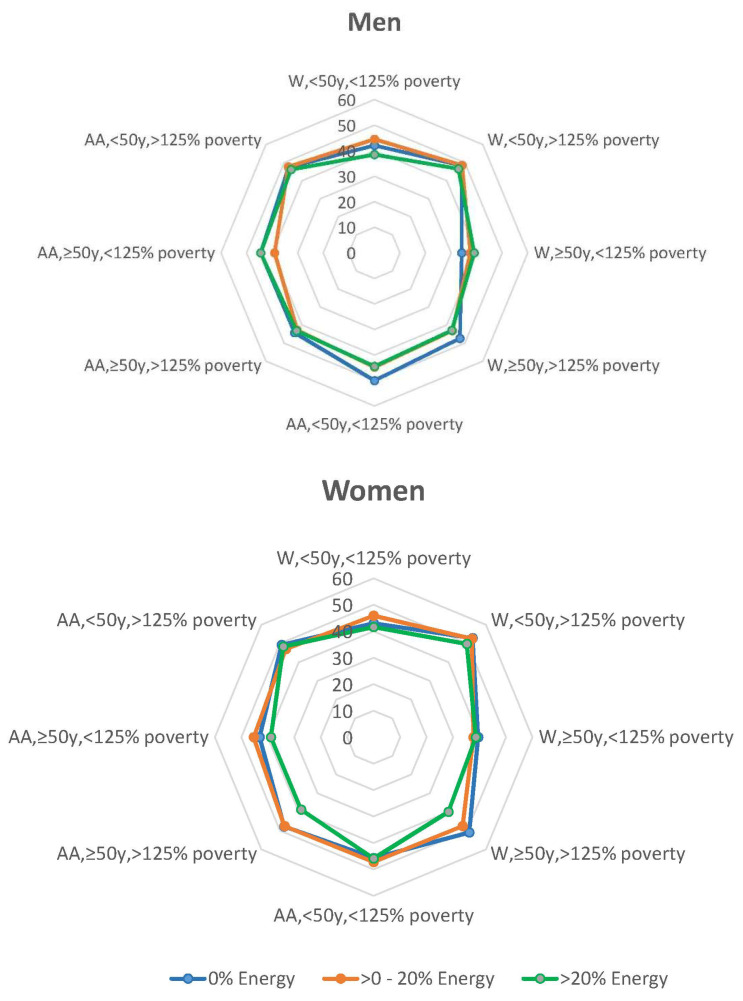
Predicted Healthy Eating Index (HEI)-2010 scores at age 50 for African American and White HANDLS study participants by initial age cohort (<50 or ≥50 years at baseline visit) and by poverty (<125% or >125% poverty threshold) for three levels of energy intake (0%, >0–20%, >20% kcal) contributed by sandwiches. HANDLS-Healthy Aging in Neighborhoods of Diversity across the Life Span. Women W—White, AA—African American.

The linear predictions of HEI-2010 scores at age 50 for all the groups based on the results of analysis of predictive margins are provided in Figure 2. Predictive margins are used to facilitate sensible interpretation of mixed models with complicated interactions. The predictive margins displayed on the radar plots allow one to compare the HEI-2010 scores for similar sex, race, and income groups but different baseline ages at the age of 50. The predicted HEI-2010 scores range from 34.15 to 52.84. It is evident from this figure that the diet quality of older African American and White women with incomes >125% of poverty status and older African American women with income <125% poverty status was better when no sandwiches were reported consumed compared to when 20% of energy intake was contributed by sandwiches. Among men, this finding was found for younger African Americans and Whites with income <125% poverty status, older African Americans with income <125% poverty, and older whites with incomes >125% poverty status. There is no identifiable pattern for diet quality when comparing scores for those who reported no sandwiches and those who consumed >0–20% of energy from sandwiches.

### 3.2. Sandwiches Consumption Patterns

Among the adults who completed both recall days for each visit, of all the sandwiches consumed, approximately 44% were eaten at lunch, 27% at dinner, 19% at breakfast and 9% as snacks. The top sandwich choices by eating occasion by age group are presented in Table 3. Egg sandwiches were the top breakfast sandwich. For lunch, cured and processed meats sandwiches ranked first and at dinner, beef sandwiches were number one. Except for snacks, the order of the top ranked sandwich choices was the same for both age groups. The only notable percentage difference for sandwich type between the groups was at dinner. While the percentage of beef sandwiches was higher for the younger group in comparison to the older group, an opposite in percentage was observed for cured and processed meats sandwiches. As for snacks, persons in the older group preferred peanut butter to beef sandwiches as the second choice for a snack. The list of sandwich type, ranked from highest to lowest frequency of report, for both age groups is presented in Table 4. As shown in this table, cured and processed meats sandwiches were the most commonly consumed type of sandwich by both age groups. 

Overall, about 65% of all the reported sandwiches by HANDLS study participants, either the ingredients for the sandwich or actual sandwich, were obtained from a store, 25% from fast food restaurants, and 4% from other restaurants. For the younger group, approximately 64% of all the sandwiches they ate came from a store and 27% from fast food restaurants (Table 4). In comparison, for persons in the older group, about 74% of sandwiches reported were obtained from stores and 22% from fast food restaurants. For each sandwich type, the source from which the sandwich or sandwich ingredients were obtained is presented in Table 4. For all sandwich types, except beef sandwiches, the most common source was a store. As shown in Table 4, beef sandwiches mostly came from restaurants—~50–53% from fast food restaurants and ~6% to 8% from other restaurants. 

## 4. Discussion

This is the first report that explores the relationship of sandwich consumption and diet quality of younger and older adults over time and describes patterns of their sandwich consumption. A dose–response relationship is not clearly evident based on HANDLS study data. Like the findings of cross-sectional analyses of other researchers who reported lower overall diet quality, measured by the HEI-2010, with sandwich consumption compared to those who reported no sandwiches [8,23], the results of our analyses on the cross-sectional exposure–diet quality relationship also found lower HEI-2010 scores for the >20% energy sandwich consumption group compared to those who did not report sandwiches. The mean HEI-2010 scores of sandwich eaters from HANDLS study were similar to those reported by An and colleagues for US adults from the WWEIA, NHANES 2003–2012 who consumed sandwiches [23].

In the HANDLS study sample, HEI-2010 scores appeared to improve over time, regardless of the sandwich energy group. Amongst the younger participants, people who did not report eating sandwiches generally had higher scores than those who consumed sandwiches that contributed >20% of daily energy intake. However, within the older group, and as people aged, sandwich consumption appeared to contribute to the rise in improvement of diet quality, resulting in HEI-2010 scores that exceeded scores of those who did not report sandwiches. To our knowledge, there have been no other articles published on the relationship of sandwich consumption and diet quality over time, limiting the comparison of our longitudinal analyses’ findings. Perhaps the older adults preferred sandwiches to cooking meals and choose healthful food items. Papanikolaou and Fulgoni suggest that the bread component and ingredients within a sandwich can be important contributors diet quality [9]. A future more in-depth examination of both these components, as well as condiments used, of the younger and older groups might provide an explanation for our findings.

Our finding of variety among top sandwich choices by eating occasion is consistent with that of Sebastian and colleagues for US adults [2]. The top two choices for breakfast, lunch, and dinner are identical. The top sandwich filling choice for snacks among HANDLS study participants was cold cuts while peanut butter was reported by US adults.

The types of sandwiches consumed by HANDLS study participants were similar to those reported by US adults, 20+ years, examined in WWEIA, NHANES 2009–2012 [2]. Cold cut sandwiches were the most commonly reported type of sandwich, followed by beef sandwiches, and then poultry sandwiches. Although the ranking differed between HANDLS study participants and WWEIA, NHANES participants, the percentages were similar for the other fillings, except fish. Fish as a primary filling was reported by 4% of US adults [2], in comparison to ~6% of younger and ~8% of older HANDLS study groups. The mean contribution of sandwiches to energy intakes based on sandwich non-reporters and consumers) for HANDLS study participants was higher than the 13% found for WWEIA, NHANES US adult population [8]. This finding was not unexpected since previous dietary pattern research using baseline data found a sandwich dietary pattern where sandwiches contributed 44% of daily energy and six of the remaining nine patterns where sandwiches ranked second in providing daily energy [24]. Fast food restaurants provided approximately the same percentage to participants in both the WWEIA, NHANES and HANDLS study (27% vs. 25%, respectively) [2].

A strength of this study is the ability to examine both cross-sectional and longitudinal exposure of dietary sandwich patterns to diet quality and the use of multiple dietary recalls to calculate HEI scores. The HANDLS study sample is racially and socioeconomically diverse, a sample that is underrepresented in the nutrition literature. The dietary collection methods used in the HANDLS study were the same as in the WWEIA, NHANES which allowed for the comparison of this urban sample to a nationally representative US population. A limitation is that dietary intakes were self-reported. Therefore, they are subject to measurement errors such as underreporting of intake and social desirability bias. It is not known if underreporting of sandwiches is similar to other foods.

## 5. Conclusions

This manuscript contributes to the literature since it is the first to describe the relationship between sandwich consumption and diet quality over time. The relationship is complex as diet quality is not only related to sandwich consumption but also age, sex, race, and income. Our results suggest that cross-sectionally, diet quality of sandwich eaters compared to non-reporters appeared to be lower but longitudinally, diet quality of older sandwich eaters compared to non-reporters seemed to improve. Yet, the HEI-2010 scores of the HANDLS study sample were reflective of low diet quality. Given sandwiches are a staple of the US diet and the primary filling is cold cuts, additional research on sandwich consumption could help to better define ways to create sandwich types that improve diet quality.

## Figures and Tables

**Table 1:** Demographic characteristics of the Healthy Aging in Neighborhoods of Diversity across the Life Span (HANDLS) study participants by visit.

Characteristic	Visit 1	Visit 2	Visit 3
	2004–2009	2009–2013	2013–2017
*n*	2177	2140	2066
Age, X ± SE	48.33 ± 0.20	53.18 ± 0.19	56.64 ± 0.20
Sex, % female	56.5%	58.8%	59.0%
Race, % AA	57.9%	61.4%	60.9%
Income, <125% poverty ^a^	42.9%	39.8%	40.7%

Abbreviations: HANDLS—Healthy Aging in Neighborhoods of Diversity across the life Span; AA—African American, SE—standard error. ^a^ <125% of the 2004 Health and Human Services poverty guidelines [12].

**Table 2:** Mean (±SE) Percentage of Daily Energy Contributed by Sandwiches (%Energy Sandwich) and Health Eating Index (HEI)-2010 Scores by Sex, Age Group, and Poverty Status for HANDLS study participants.

	Women				Men			
Age at Baseline Visit	<50 Years		≥50 Years		<50 Years		≥50 Years	
	*N*	% Energy Sandwich	*N*	% Energy Sandwich	*N*	% Energy Sandwich	*N*	% Energy Sandwich
<125% Poverty ^a^								
White	295	19.21 ± 0.92	223	18.12 ± 1.03	168	19.75 ± 1.26	126	25.31 ± 1.56
African American	700	17.65 ± 0.60	412	17.68 ± 0.84	436	19.79 ± 0.76	266	19.10 ± 0.99
>125% Poverty								
White	509	15.41 ± 0.69	455	16.20 ±0.73	400	20.14 ± 0.80	373	18.40 ± 0.85
African American	584	17.65 ± 0.64	531	17.20 ± 0.68	499	20.81 ± 0.80	406	19.73 ± 0.85
		**HEI-2010**		**HEI-2010**		**HEI-2010**		**HEI-2010**
<125% Poverty								
White	295	41.64 ± 0.66	223	45.66 ± 0.84	168	40.09 ± 0.81	126	40.48 ± 0.96
African American	700	44.13 ± 0.41	412	47.50 ± 0.58	436	43.50 ± 0.46	266	43.32 ± 0.64
>125% Poverty								
White	509	47.97 ± 0.64	455	49.08 ± 0.66	400	44.90 ± 0.63	373	47.49 ± 0.68
African American	584	45.73 ± 0.45	531	50.11 ± 0.54	499	44.67 ± 0.47	406	46.91 ± 0.57

Abbreviations: HANDLS—Healthy Aging in Neighborhoods of Diversity across the Life Span; *N*—number of observations; SE—standard error. HEI—Healthy Eating Index. ^a^ Poverty status dichotomized into <125% and >125% of the 2004 Health and Human Services poverty guidelines [12].

**Table 3:** Top three types of sandwiches consumed by eating occasion by age of HANDLS study participants (*n* = 1213) ^a^.

Age at Baseline Visit <50 Years	%	Age at Baseline Visit ≥ 50 Years	%
Breakfast		Breakfast	
Egg	46.1	Egg	45.3
Cured and processed meats	32.1	Cured and processed meats	33.0
Poultry	5.2	Poultry	5.1
Lunch		Lunch	
Cured and processed meats	32.7	Cured and processed meats	31.6
Beef	23.7	Beef	20.7
Poultry	16.3	Poultry	14.4
Dinner		Dinner	
Beef	40.9	Beef	31.1
Cured and processed meats	18.4	Cured and processed meats	24.2
Poultry	14.3	Poultry	14.2
Snack		Snack	
Cured and processed meats	29.8	Cured and processed meats	28.1
Beef	16.0	Peanut Butter	15.7
Poultry	14.5	Poultry	14.7

Abbreviations: HANDLS—Healthy Aging in Neighborhoods of Diversity across the Life Span. ^a^ Participants completed 2 dietary recalls for 3 study visits between 2004 and 2017.

**Table 4:** Type of sandwich reported consumed and where sandwich obtained by HANDLS study participants by age (*n* = 1213) ^a^.

Age at Baseline Visit <50 Years					Age at Baseline Visit ≥ 50 Years				
Sandwich Type	%	Where Obtained, %			Sandwich Type	%	Where Obtained, %		
		Fast Food Restaurant	Other Restaurant ^b^	Store ^c^			Fast Food Restaurant	Other Restaurant ^b^	Store ^c^
Cured and processed meats	28.7	17.5	1.6	80.8	Cured and processed meats	29.9	12.9	2.7	84.4
Beef	22.9	53.2	5.9	40.8	Beef	18.5	50.2	7.5	42.3
Poultry	14.4	32.2	6.6	61.2	Poultry	13.4	24.8	3.4	71.9
Egg	9.6	35.2	4.6	60.2	Egg	9.2	29.5	3.1	67.4
Hot dogs	8.7	5.1	1.0	93.9	Hot dogs	8.5	5.8	9.7	92.8
Fish	6.4	33.5	2.3	64.2	Fish	8.4	23.9	7.8	68.3
Peanut Butter	3.8	0	0	100	Cheese	5.1	9.7	3.2	87.1
Cheese	3.7	16.9	2.4	79.8	Peanut Butter	3.8	0	0	100
Pork	1.0	2.9	0	97.1	Pork	1.8	2.3	2.3	95.3
Vegetable and fruit	0.9	13.3	10.0	76.7	Vegetable and fruit	1.4	15.2	3.0	81.8
Mean		26.9	3.5	63.7	Mean		22.4	4.0	73.6

^a^ Participants completed two dietary recalls for three study visits between 2004–2017. ^b^ Other restaurant included: restaurant with waiter/waitress, bar/tavern, restaurant no information. ^c^ Store includes grocery store, convenience store, store—no information. Abbreviations: HANDLS—Healthy Aging in Neighborhoods of Diversity across the Life Span.

## Supplementary Materials

The following are available online at https://www.mdpi.com/2072-6643/12/9/2807/s1, Table S1: Mixed Model Regression Results of Relationship of Energy from Sandwiches with Healthy Eating Index-2010 scores as outcome, Table S2: Change in Healthy Eating Index-2010 scores based on mixed-effect regression analyses.
