# Supplementary material for: The Relationship of Diet Quality with Proportion of Daily Energy Contributed by Sandwiches Varies by Age over Adulthood in Racially and Socioeconomically Diverse Adults
Source: Nutrients. 2020 Sep 13;12(9):2807. doi: 10.3390/nu12092807 (PMC7551748; doi:10.3390/nu12092807)
Supplement: Supplementary file 1 [file nutrients-12-02807-s001.pdf]

Supplementary methods
Mixed model, testing multiple parameters in 5-wayears interaction

cd "D:\...\DATA"

use 2020–04–17-Q2,clear

**TotalHEIScore ~ (SandEnerGrp + cenAge + Sex + Race + PovStat + Age0 Grp) ^5 + (cenAge | HNDid)

*****************************FINAL CODE: TWO wayears Output*****************

xtmixed TotalHEIScore SandEnerGrp##c.cenAge##Sex##Race##PovStat##Age0 Grp || HNDid: cenAge

Main effect byears each group interacted with sandwich consumption

margins SandEnerGrp#Sex, dyearsdx(_cons)

margins SandEnerGrp#Race, dyearsdx(_cons)

margins SandEnerGrp#PovStat, dyearsdx(_cons)

margins SandEnerGrp#Age0 Grp, dyearsdx(_cons)

Slopes byears each group interacted with sandwich consumption

margins SandEnerGrp#Sex, dyearsdx(c.cenAge)

margins SandEnerGrp#Race, dyearsdx(c.cenAge)

margins SandEnerGrp#PovStat, dyearsdx(c.cenAge)

margins SandEnerGrp#Age0 Grp, dyearsdx(c.cenAge)

****************FINAL CODE: THREE WAY OUTPUT BY RACE*******************

cd "D:\...\DATA"

use 2020–04–17-Q2,clear

xtmixed TotalHEIScore SandEnerGrp##c.cenAge##Sex##Race##PovStat##Age0 Grp || HNDid: cenAge

Main effect byears each group interacted with sandwich consumption

margins SandEnerGrp#Sex##Race, dyearsdx(_cons)

margins SandEnerGrp#PovStat##Race, dyearsdx(_cons)

margins SandEnerGrp#Age0 Grp##Race, dyearsdx(_cons)

Slopes byears each group interacted with sandwich consumption

margins SandEnerGrp#Sex##Race, dyearsdx(c.cenAge)

margins SandEnerGrp#PovStat##Race, dyearsdx(c.cenAge)

margins SandEnerGrp#Age0 Grp##Race, dyearsdx(c.cenAge)

****************FINAL CODE: FOUR WAY OUTPUT******************

```
cd “D:\...\DATA”

use 2020–04−17-Q2,clear

xtmixed TotalHEIScore SandEnerGrp##c.cenAge##Sex##Race##PovStat##Age0 Grp || HNDid: cenAge

**Main effect byears each group interacted with sandwich consumption**

margins SandEnerGrp#Sex##Race##PovStat, dyearsdx(_cons)

margins SandEnerGrp#Age0 Grp##Race##PovStat, dyearsdx(_cons)

**Slopes byears each group interacted with sandwich consumption**

margins SandEnerGrp#Sex##Race##PovStat, dyearsdx(c.cenAge)

margins SandEnerGrp#Age0 Grp##Race##PovStat, dyearsdx(c.cenAge)

******************FINAL CODE: FIVE WAY OUTPUT*********************

cd “D:\...\DATA”

use 2020–04−17-Q2,clear

xtmixed TotalHEIScore SandEnerGrp##c.cenAge##Sex##Race##PovStat##Age0 Grp || HNDid: cenAge

**Main effect byears each group interacted with sandwich consumption**

margins SandEnerGrp#Sex##Race##PovStat##Age0 Grp, dyearsdx(_cons)

**Slopes byears each group interacted with sandwich consumption**

margins SandEnerGrp#Sex##Race##PovStat##Age0 Grp, dyearsdx(c.cenAge)
```

Table S1. Mixed Model Regression Results of Relationship of Energy from Sandwiches with Healthy Eating Index−2010 scores as outcome.

Variable	Coefficient	*p*
Intercept	51.33	<0.001
Sandwich En > 0–20	−0.28	0.85
Sandwich En > 20	−3.30	**0.02**
cenAge	5.33	**<0.001**
SexMen	−3.70	0.07
RaceAA	−3.19	**0.04**
PovStatBelow	−8.34	**<0.001**
AgeGroup ≥ 50 years	−0.14	0.95
Sandwich En > 0–20:cenAge	−0.23	0.89
Sandwich En > 20:cenAge	0.88	0.62
Sandwich En > 0–20:SexMen	−0.26	0.92
Sandwich En > 20:SexMen	1.02	0.66
Sandwich En > 0–20:RaceAA	−1.76	0.37
Sandwich En > 20:RaceAA	2.13	0.25
Sandwich En > 0–20:PovStatBelow	1.24	0.62
Sandwich En > 20:PovStatBelow	1.37	0.57
Sandwich En > 0–20: AgeGroup ≥ 50 years	−4.22	0.15
Sandwich En > 20: AgeGroup ≥ 50 years	−10.21	**0.001**

cenAge:SexMen	−4.03	0.09
cenAge:RaceAA	−1.66	0.37
cenAge:PovStatBelow	−5.80	**0.02**
cenAge:AgeGroup ≥ 50 yearsear	−5.66	**0.01**
SexMen:RaceAA	2.33	0.37
SexMen:PovStatBelow	2.26	0.51
SexMen:AgeGroup ≥ 50 yearsear	−1.23	0.74
RaceAA:PovStatBelow	5.69	**0.03**
RaceAA:AgeGroup ≥ 50 years	−1.41	0.67
PovStatBelow:AgeGroup ≥ 50 years	−5.94	0.17
Sandwich En > 0–20:cenAge:SexMen	3.72	0.20
Sandwich En > 20:cenAge:SexMen	2.36	0.41
Sandwich En > 0–20:cenAge:RaceAA	−0.53	0.82
Sandwich En > 20:cenAge:RaceAA	0.59	0.80
Sandwich En > 0–20:cenAge:PovStatBelow	7.44	**0.02**
Sandwich En > 20:cenAge:PovStatBelow	1.77	0.53
Sandwich En > 0–20:cenAge: AgeGroup ≥ 50 years	3.85	0.16
Sandwich En > 20:cenAge: AgeGroup ≥ 50 years	7.40	**0.01**
Sandwich En > 0–20:SexMen:RaceAA	2.35	0.45
Sandwich En > 20:SexMen:RaceAA	−1.52	0.60
Sandwich En > 0–20:SexMen:PovStatBelow	1.43	0.72
Sandwich En > 20:SexMen:PovStatBelow	−2.33	0.54
Sandwich En > 0–20:SexMen:AgeGroup ≥ 50 years	−0.13	0.98
Sandwich En > 20:SexMen:AgeGroup ≥ 50 years	8.18	0.08
Sandwich En > 0–20:RaceAA:PovStatBelow	1.30	0.67
Sandwich En > 20:RaceAA:PovStatBelow	−1.04	0.72
Sandwich En > 0–20:RaceAA:AgeGroup ≥ 50 years	6.40	0.13
Sandwich En > 20:RaceAA:AgeGroup ≥ 50 years	0.95	0.82
Sandwich En > 0–20:PovStatBelow:AgeGroup ≥ 50 years	1.08	0.83
Sandwich En > 20:PovStatBelow:AgeGroup ≥ 50 years	11.95	**0.02**
cenAge:SexMen:RaceAA	2.90	0.33
cenAge:SexMen:PovStatBelow	7.09	0.07
cenAge:SexMen:AgeGroup ≥ 50 years	7.86	**0.02**
cenAge:RaceAA:PovStatBelow	3.24	0.26
cenAge:RaceAA:AgeGroup ≥ 50 years	6.32	**0.03**
cenAge:PovStatBelow:AgeGroup ≥ 50 years	14.67	**<0.001**
SexMen:RaceAA:PovStatBelow	2.27	0.58
SexMen:RaceAA:AgeGroup ≥ 50 years	−0.95	0.85
SexMen:PovStatBelow:AgeGroup ≥ 50 years	−0.51	0.95
RaceAA:PovStatBelow:AgeGroup ≥ 50 years	3.33	0.53
Sandwich En > 0–20:cenAge:SexMen:RaceAA	−2.33	0.52
Sandwich En > 20:cenAge:SexMen:RaceAA	−2.55	0.47
Sandwich En > 0–20:cenAge:SexMen:PovStatBelow	−10.34	**0.02**
Sandwich En > 20:cenAge:SexMen:PovStatBelow	−7.08	0.10
Sandwich En > 0–20:cenAge:SexMen:AgeGroup ≥ 50 years	−5.26	0.20
Sandwich En > 20:cenAge:SexMen:AgeGroup ≥ 50 years	−10.22	**0.01**
Sandwich En > 0–20:cenAge:RaceAA:PovStatBelow	−3.29	0.37
Sandwich En > 20:cenAge:RaceAA:PovStatBelow	0.18	0.96
Sandwich En > 0–20:cenAge:RaceAA:AgeGroup ≥ 50 years	−4.59	0.22
Sandwich En > 20:cenAge:RaceAA:AgeGroup ≥ 50 years	−3.22	0.38
Sandwich En > 0–20:cenAge:PovStatBelow:AgeGroup ≥ 50 years	−9.33	**0.04**
Sandwich En > 20:cenAge:PovStatBelow:AgeGroup ≥ 50 years	−10.95	**0.01**

Sandwich En > 0–20:SexMen:RaceAA:PovStatBelow	−8.57	0.07
Sandwich En > 20:SexMen:RaceAA:PovStatBelow	−1.45	0.75
Sandwich En > 0–20:SexMen:RaceAA:AgeGroup ≥ 50 years	−3.65	0.57
Sandwich En > 20:SexMen:RaceAA:AgeGroup ≥ 50 years	1.65	0.79
Sandwich En > 0–20:SexMen:PovStatBelow:AgeGroup ≥ 50 years	3.74	0.66
Sandwich En > 20:SexMen:PovStatBelow:AgeGroup ≥ 50 years	−2.16	0.80
Sandwich En > 0–20:RaceAA:PovStatBelow:AgeGroup ≥ 50 years	−0.89	0.89
Sandwich En > 20:RaceAA:PovStatBelow:AgeGroup ≥ 50 years	−6.44	0.33
cenAge:SexMen:RaceAA:PovStatBelow	−2.25	0.61
cenAge:SexMen:RaceAA:AgeGroup ≥ 50 years	−6.57	0.13
cenAge:SexMen:PovStatBelow:AgeGroup ≥ 50 years	−15.16	**0.004**
cenAge:RaceAA:PovStatBelow:AgeGroup ≥ 50 years	−9.44	**0.03**
SexMen:RaceAA:PovStatBelow:AgeGroup ≥ 50 years	1.83	0.83
Sandwich En > 0–20:cenAge:SexMen:RaceAA:PovStatBelow	3.06	0.56
Sandwich En > 20:cenAge:SexMen:RaceAA:PovStatBelow	2.72	0.58
Sandwich En > 0–20:cenAge:SexMen:RaceAA:AgeGroup ≥ 50 years	5.72	0.27
Sandwich En > 20:cenAge:SexMen:RaceAA:AgeGroup ≥ 50 years	4.10	0.41
Sandwich En > 0–20:cenAge:SexMen:PovStatBelow:AgeGroup ≥ 50 years	10.93	**0.04**
Sandwich En > 20:cenAge:SexMen:PovStatBelow:AgeGroup ≥ 50 years	13.11	**0.008**
Sandwich En > 0–20:cenAge:RaceAA:PovStatBelow:AgeGroup ≥ 50 years	3.99	0.45
Sandwich En > 20:cenAge:RaceAA:PovStatBelow:AgeGroup ≥ 50 years	3.38	0.50
Sandwich En > 0–20:SexMen:RaceAA:PovStatBelow:AgeGroup ≥ 50 years	−3.89	0.70
Sandwich En > 20:SexMen:RaceAA:PovStatBelow:AgeGroup ≥ 50 years	−5.12	0.59
cenAge:SexMen:RaceAA:PovStatBelow:AgeGroup ≥ 50 years	5.63	0.22

Abbreviations: AA – African American; cenAge – centered age; En-energy; PovStatBelow – poverty status below (< 125% poverty)

Table S2. Change in Healthy Eating Index−2010 scores based on mixed-effect regression analyses.

Group	Slope	SE	*p*
**Men**			
0 energy from sandwich			
**African American**			
<50 years, <125% poverty	4.93	1.65	**0.003**
<50 years, >125% poverty	2.45	1.45	0.092
≥50 years, <125% poverty	2.19	2.44	0.370
≥50 years, >125% poverty	4.57	1.95	**0.019**
**White**			
<50 years, <125% poverty	2.35	2.73	0.389
<50 years, >125% poverty	1.97	2.04	0.334
≥50 years, <125% poverty	5.30	5.33	0.320
≥50 years, >125% poverty	3.34	1.97	0.091
>0–20% energy from sandwich			
**African American**			
<50 years, <125% poverty	2.47	1.21	**0.042**
<50 years, >125% poverty	3.18	1.33	**0.017**
≥50 years, <125% poverty	5.18	2.17	**0.017**
≥50 years, >125% poverty	4.84	1.93	**0.012**

**White**			
<50 years, <125% poverty	3.28	1.99	0.099
<50 years, >125% poverty	5.04	1.52	**0.001**
≥50 years, <125% poverty	5.33	3.23	0.099
≥50 years, >125% poverty	5.44	1.85	**0.003**
>20% energy from sandwich			
**African American**			
<50 years, <125% poverty	3.68	1.17	**0.002**
<50 years, >125% poverty	3.82	1.13	**0.001**
≥50 years, <125% poverty	5.08	1.85	**0.006**
≥50 years, >125% poverty	3.65	1.53	**0.017**
**White**			
<50 years, <125% poverty	0.77	1.81	0.672
<50 years, >125% poverty	4.49	1.35	**0.001**
≥50 years, <125% poverty	1.06	2.83	0.707
≥50 years, >125% poverty	4.00	1.66	**0.016**
**Women**			
0 energy from sandwich			
**African American**			
<50 years, <125% poverty	1.08	1.18	0.362
<50 years, >125% poverty	3.84	1.30	**0.003**
≥50 years, <125% poverty	7.11	1.63	**<0.001**
≥50 years, >125% poverty	4.26	1.62	**0.008**
**White**			
<50 years, <125% poverty	−0.16	2.05	0.937
<50 years, >125% poverty	5.59	1.40	**<0.001**
≥50 years, <125% povert years	8.25	2.50	**0.001**
≥50 years, >125% poverty	−0.24	1.67	0.886
>0–20% energy from sandwich			
**African American**			
<50 years, <125% poverty	4.59	1.05	**<0.001**
<50 years, >125% poverty	3.12	1.20	**0.009**
≥50 years, <125% poverty	4.36	1.75	**0.013**
≥50 years, >125% poverty	2.70	1.66	0.105
**White**			
<50 years, <125% poverty	7.07	1.92	**<0.001**
<50 years, >125% poverty	5.35	1.22	**<0.001**
≥50 years, <125% poverty	10.08	2.29	**<0.001**
≥50 years, >125% poverty	3.40	1.55	**0.028**
>20% energy from sandwich			
**African American**			
<50 years, <125% poverty	4.67	0.95	**<0.001**
<50 years, >125% poverty	5.16	1.10	**<0.001**
≥50 years, <125% poverty	6.84	1.49	**<0.001**
≥50 years, >125% poverty	10.03	1.45	**<0.001**
**White**			
<50 years, <125% poverty	2.21	1.38	0.109
<50 years, >125% poverty	6.46	1.29	**<0.001**
≥50 years, <125% poverty	7.85	2.06	**<0.001**
≥50 years, >125% poverty	7.73	1.66	**<0.001**
